# Novel Module-Based Membrane Reactor Design Approach for Improved Operability Performance

**DOI:** 10.3390/membranes11020157

**Published:** 2021-02-23

**Authors:** Brent A. Bishop, Fernando V. Lima

**Affiliations:** Department of Chemical and Biomedical Engineering, West Virginia University, Morgantown, WV 26506, USA; babishop@mix.wvu.edu

**Keywords:** process intensification, modular systems, membrane reactors

## Abstract

This work aims to address the design and control challenges caused by the integration of phenomena and the loss of degrees of freedom (DOF) that occur in the intensification of membrane reactor units. First, a novel approach to designing membrane reactor units is proposed. This approach consists of designing smaller modules based on specific phenomena such as heat exchange, reactions, and mass transport and combining them in series to produce the final modular membrane-based unit. This approach to designing membrane reactors is then assessed using a process operability analysis for the first time to maximize the operability index, as a way of quantifying the operational performance of intensified processes. This work demonstrates that by designing membrane reactors in this way, the operability of the original membrane reactor design can be significantly improved, translating to an improvement in achievability for a potential control structure implementation.

## 1. Introduction

The challenge of reduced degrees of freedom (DOF) in intensified processes is one that has been identified in literature as early as 2003 [1] and has continued to be studied and discussed to this day [2,3,4,5]. To understand this body of research, it is important to categorize the DOF into two main categories. The first are the design DOF, and they include all the equipment design parameters such as length, diameter, membrane thickness, mass of catalyst, etc. The others are the operational DOF which encompass the operating conditions and manipulated variables used to control the process.

Reported literature [1] has identified the reduction in DOF as being caused by the coupling of certain variables when a process becomes intensified. Using the example of membrane systems, in a conventional process with separate reactor and membrane separation units, there is freedom to choose the length for each piece of equipment, whereas in the intensified case, i.e., a membrane reactor, these lengths become equal, and therefore a loss in a design DOF occurs. There are also reductions in the operational DOF due to coupling of heat duty, catalyst-related reaction rates, and permeation rates. Because these phenomena are occurring simultaneously, they become interdependent with respect to each other.

Although the challenge of reduced DOF in the control of intensified processes has been identified, the literature remained mostly hypothetical and speculative when it came to proposing solutions. Due to the DOF reduction occurring because of the design decision to intensify the process, some claimed a different approach to process synthesis where “process design, operation, and control should be considered simultaneously” was necessary to address this challenge [5]. The reported studies thus show there is an existing gap in the literature regarding solutions for process design and control changes when a process is intensified. 

For almost as long as the challenge of DOF reduction has been know, the literature has consisted of empirical arguments for why this problem occurs and was not until recently (2017) that a more rigorous justification was provided [2]. The modular technology research within the process systems engineering community has specifically identified this and other control challenges in general as an important step for the advancement of modular technologies [3,4]. Hence, there is a need to develop a theoretical basis for understanding the challenge of DOF reduction in modular intensified systems and what steps can be taken to improve the performance of these systems. 

In the chemical industry, a modular plant is a unit where “the process equipment, instrumentation, valves, piping components, and electrical wiring are mounted within a structural steel framework” [6]. In even simpler terms, the main focus of modularity with respect to the chemical industry is to scale down traditionally large processes in such a way that they can fit inside a structure to be transported via a flatbed truck [7]. This allows for them to be transported to various sites such as natural gas or oil wells and deployed more easily without the need of pipelines. This idea of reducing the size of chemical processes runs counter to the concept of “economies of scale” which states that, in general, larger chemical facilities are more profitable (due to many factors, but most notably cheaper per-unit capital cost of larger equipment). Process intensification attempts to address this problem by using technologies that on these modular scales, make the chemical process more efficient. 

This cheaper-per-unit capital versus higher efficiency tradeoff is not the only tradeoff to consider when designing modular and traditional plants. This work focuses on the operability and control challenges that are caused by this change in intensified designs. If an engineer is given a nominal operating condition, a membrane reactor designed for that nominal condition will intuitively outperform the unit operations-based design at that same nominal condition in terms of efficiency and most performance metrics. However, if there is a deviation from that nominal condition, say, the process needed to be scaled up or moved to another location, then the unit operations-based design (when membrane and reactor are used in separate) will intuitively outperform the membrane reactor at the new condition. This is because when reactions, separations, and heat exchange are performed independently rather than simultaneously such as in a membrane reactor, each phenomenon can be independently manipulated to produce the desired output given the change in the operating condition. 

That said, a paradox the process intensification community faces is that smaller units need to be produced to allow for transportation from, say, one location/natural gas well site to another. This reduction in size necessitates using technology-like membrane reactors to increase the efficiency of the unit to maintain profitability. However, by choosing a purely membrane-reactor design, the unit can no longer operate as effectively as required because it cannot adapt to the different operating conditions at the various locations/well sites. Therefore, unique modular membrane reactors would have to be designed for each well site’s conditions and the source of the profitability for producing modular equipment in the first place is lost. This suggests that a new perspective on what “modular” means in the chemical industry is desired. Specifically, a definition that balances the performance of the membrane reactor and the adaptability of the unit operations-based approach. 

The traditional definition of modularity in the chemical industry contrasts with other industries such as the smartphone, computer, and automobile industries who emphasize the customizability, upgradeability, and low cost-of-repair for their own modular technologies [8,9,10]. If this definition of modularity were applied to the chemical industry, the emphasis would switch from producing modular process units and instead thinking of modular equipment that can be customized, upgraded, or repaired without having to buy a completely new piece of equipment. Switching to an approach as this could further reduce costs for modular technologies by mass producing each module, while also allowing for equipment to be designed for a specific application. However, the increase in possible combinations of module-based designs is a challenge in and of itself. With each additional module added to the design, four additional options (heat exchanger, membrane separator, reactor, or membrane reactor) would be introduced leading to a combinatorics problem that grows exponentially. When scaling a process down using this concept, the chemical industry should consider new approaches to process design other than traditional methods [11]. In this research, the idea of module-based equipment as opposed to modular plants will be explored as a potential solution for cutting costs and addressing the DOF reduction challenge. This modular equipment will be simulated using the AVEVA Process Simulation membrane reactor model developed by our team and that is now available in the most recent addition of AVEVA Process Simulation, version 5.0.

Thus, in this work, a block-based phenomena approach to modeling modular membrane systems developed in previous work [12] is extended and applied for the first time to improve the operability index of the proposed design. Other groups have also investigated operability concepts applied to block-based phenomena systems, notably reactive separation systems [13,14,15]. However, the current body of literature has focused primarily on the inclusion of a flexibility analysis as part of an economic optimization, whereas this work is focused purely on the improvement of the operability of a membrane reactor system quantified by the operability index (OI). This work chooses the maximization of OI as its objective instead for solving the DOF challenge because, as stated earlier, the integration of process design, operation, and control should be considered simultaneously [5]. Determining the OI of a design is extremely useful for addressing this need for such simultaneous considerations because it has been proven that the OI is independent of the eventual control structure selected [16], providing a measure of improvement in a design’s performance for control implementation. 

The rest of the paper is organized as follows: First, background is provided about the proposed module-based design approach to membrane reactor systems, the method used for simulating these systems, and process operability concepts. Process operability analysis is then systematically applied to these module-based designs to gain insights to how this design approach can improve the performance of a base case membrane reactor. Lastly, the paper provides conclusion and outline some directions for future work.

## 2. Module-Based Design, Simulation, and Process Operability Background

The following subsections provide a brief background into the concepts of modularity and operability as they will be defined and used in this research.

### 2.1. Modular Design Background

**Figure 1:**
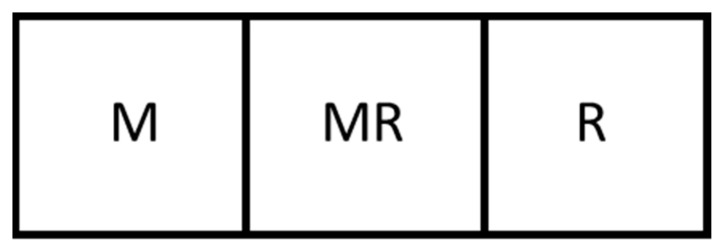
An example arrangement of three modules to make a larger intensified unit. Here a membrane separator (M), a membrane reactor (MR), and a reactor (R) are combined.

As the literature has identified, much of the problem with the loss of DOF when optimizing the design and operation of intensified process units such as membrane reactors is due to the coupling of design parameters/dimensions and physical phenomena. This work addresses this challenge by introducing a novel design method for membrane reactor units that allows for the partial decoupling of such parameters and phenomena. Rather than designing a single membrane reactor unit, this work proposes designing units through the assembly of smaller, phenomena-specific modular units. For example, a membrane reactor unit could be assembled by combining a membrane module, a membrane reactor module, and a reactor module in an arrangement as in Figure 1.

By designing membrane reactor units in this way, there is now the freedom to choose the lengths of membrane and catalyst sections as well as introduce sections where the different phenomena are not occurring simultaneously. However, this approach also introduces important new design considerations. First, the construction of small process units goes against the normal paradigm of economies of scale which finds that processes on larger scales are more economical. For a modular system to be profitable, it must be more efficient than a conventional process and should be a design that can be mass produced. This means each heat exchanger, membrane separator, reactor, and membrane reactor modules would ideally be constrained to, for example, a certain size, length, or number of tubes to reduce costs.

One of the major benefits of the design approach presented here is that individual modules can be mass produced and combined in a number of permutations to meet a desired objective given a certain set of potential inputs (such as the set of all inputs from all potential well sites the unit will operate considering the shale gas utilization problem as an example). To simulate these module-based designs, a novel simulation approach using block-based phenomena modeling was developed in previous work [12] and is summarized below.

### 2.2. Block-Based Phenomena Simulation

**Figure 2:**
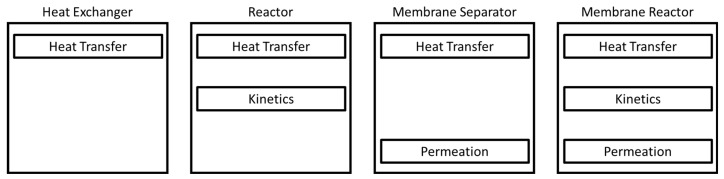
A breakdown of the individual phenomena that take place in each unit operation.

To simulate the type of modular equipment being proposed in this work, the modeling approach has to allow for multiple unit operations to be simulated by the same model without making topological changes in the simulation space. The method proposed here is to build each unit operation by including or excluding the phenomena that occur within them. An easy analogy for this is to view the individual phenomena as modules for a more complex unit operation as illustrated in Figure 2.

**Figure 3:**
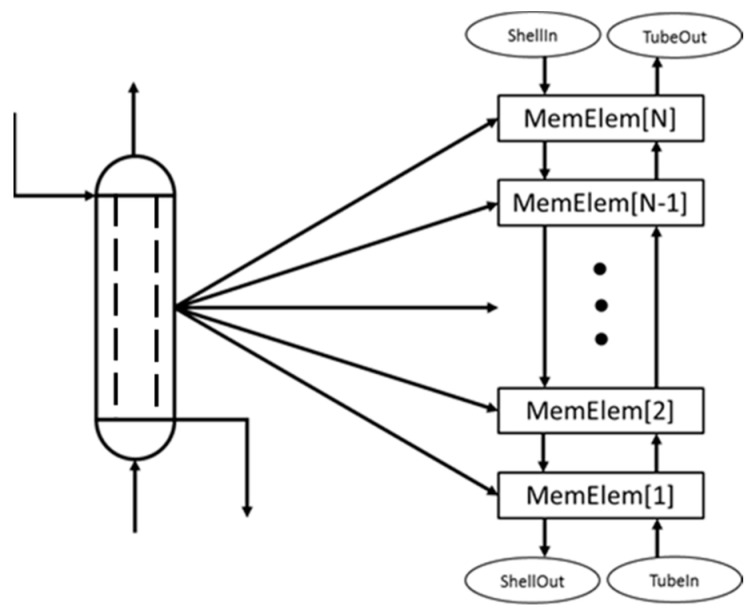
Each piece of modular equipment consists of individual modules in series. These modules have been fully developed in AVEVA Process Simulation and can be a heat exchanger, reactor, membrane separator, or membrane reactor.

The smaller modules that may include such phenomena are connected in series to simulate the full-scale modular equipment as seen in Figure 3.

With this model structure in place, the last required step is the inclusion of a “contact” variable. Because AVEVA Process Simulation is an equation-oriented environment, adding or removing membrane or catalyst discretely would likely cause the model to become unsolved, especially when the model is running in countercurrent, non-isothermal operation. This necessitates converting the discrete design space into a continuous one using the concept of a contact variable. For example, take the component mass balance through a thin slice of the membrane reactor:

(1) $$F_{i,in}+V_{r}r_{i}-A_{p}J_{i}=F_{i,out}$$

in which *F_i_* is the component molar flows in and out of the slice, *V_r_* is the volume where reaction takes place, *r_i_* is the component reaction rate, *A_p_* is the area of permeation, and *J_i_* is the component flux through the membrane. The contact variables for reaction and permeation, *c_r_* and *c_p_*, can be added as follows:

(2) $$F_{i,in}+V_{r}r_{i}*c_{r}-A_{p}J_{i}*c_{p}=F_{i,out}$$

The inclusion of these variables in the mass and energy balances of the model allows for a continuous transition from having catalyst and membrane (contact values of 1) to designs that are missing one or the other or both (contact values of 0). This feature is also beneficial because it converts a potential optimization problem from a mixed-integer nonlinear programming problem (MINLP) to just a nonlinear programming problem (NLP), allowing for the utilization of gradient-based solving techniques.

In a membrane reactor, there are three factors that contribute to changes in temperature: reactions, heat duty, and the Joules-Thomson effect as material permeates from one side of the membrane to the other. Considering a thin control volume along the length of the membrane reactor, these three effects can be modeled with the following energy balances:

Energy balance, tube:

(3) $$0=\frac{d\left(F_{t}H_{t}\right)}{dz}+\underset{i}{\sum }J_{i}\pi d_{t}\int_{p_{t}}^{p_{s}}{\left(\frac{\partial H_{i}}{\partial p}\right)}_{T_{i}}dp+U\pi d_{t}\left(T_{t}-T_{s}\right)$$

Energy balance, shell:

(4) $$0=\frac{d\left(F_{s}H_{s}\right)}{dz}+\underset{i}{\sum }J_{i}\pi d_{t}\int_{p_{t}}^{p_{s}}{\left(\frac{\partial H_{i}}{\partial p}\right)}_{T_{i}}dp+U\pi d_{t}\left(T_{t}-T_{s}\right)$$

in which the central term accounts for the Joule-Thomson effect caused by material leaving or entering each side through the membrane. In addition, *p_t_* is the total tube pressure, *p_s_* is the total shell pressure, and ${\left(\frac{\partial H_{i}}{\partial p}\right)}_{T_{i}}$ is the isothermal Joule-Thomson coefficient for species *i* at constant temperature $T_{i}$ (where $T_{i}$ is the temperature of the side species *i* originated). The final term on the right accounts for the energy gained or lost through heat transfer across the tube where U is the overall heat transfer coefficient and is a function of the tube and shell side flow rates and $d_{t}$ is the diameter of the tube. The inclusion of making U a function of the flow rates is a new addition to the model from previous work [12].

Lastly, the study is performed assuming steady state using pressure-driven flow calculations, necessitating rigorous pressure drop calculations. The process side, which is a packed bed, is modeled using the Ergun equation (5) while the sweep gas side uses the Colbrooke equation (6) for modeling the pressure drop.

Ergun Equation, tube:

(5) $$\frac{dp_{t}}{dz}=\frac{150\mu }{D_{p}{}^{2}}\frac{{\left(1-\epsilon \right)}^{2}}{\epsilon^{3}}v_{s}+\frac{1.75\rho }{D_{p}}\frac{\left(1-\epsilon \right)}{\epsilon^{3}}v_{s}\left|v_{s}\right|$$

in which *μ* is the fluid’s viscosity, *D_p_* is the catalyst particle diameter, ε is the void fraction of the catalyst, *v_s_* is the superficial velocity of the fluid, and ρ is the fluid density.

Colebrook Equation, shell:

(6) $$\frac{dp_{s}}{dz}=\frac{64f\mu v_{s}}{2D_{h}{}^{2}}$$

where *D_h_* is the hydraulic diameter of the shell side and f is the Darcy friction factor and can be solved with the implicit formula:

(7) $$\frac{1}{\sqrt{f}}=-2\log\left(\frac{\epsilon }{3.7D_{h}}+\frac{2.51}{Re\sqrt{f}}\right)$$

### 2.3. Process Operability Background

Set-point operability is used in this research to measure the performance of each modular equipment design, as defined in the operability analysis and concepts for square systems [17]. The mapping of inputs ($u\in {\mathbb{R}}^{m}$) of a model (**M**) to its outputs ($y\in {\mathbb{R}}^{p}$) can be formulated the following way:

(8) $$M=\{\begin{array}{c} \dot{x}=f\left(x,u\right)\\ y=g\left(x,u\right)\\ \;h_{1}\left(\dot{x},x,y,\dot{u},u\right)=0\\ \;h_{2}\left(\dot{x},x,y,\dot{u},u\right)\geq 0 \end{array}$$

in which $x\in {\mathbb{R}}^{n}$ are the state variables and $h_{1}$ and $h_{2}$ are equality and inequality process constraints, respectively. In addition, $\dot{x}$ and $\dot{u}$ represent time derivatives associated with x and u, respectively, and f and g are nonlinear process maps.

Using the operability input-output mapping concepts, there are three sets of inputs and outputs that are important in the analysis performed in this article:

Available Input Set (AIS): The set of all operational inputs or manipulated variables that are available to produce changes to the outputs of the process and is defined as:

(9) $$AIS=\left\{u|u_{i}^{min}\leq u_{i}\leq \;u_{i}^{max};1\leq i\leq m\right\}$$

Because the AIS would include the set of every combination of possible control moves that an undetermined control structure could impose on the system, this analysis is independent of the defined control structure. This feature makes operability especially appealing for the design of modular membrane systems for addressing the DOF reduction challenge.

Desired Output Set (DOS): This set represents the region of operation that is desired for a given process and is defined as:

(10) $$DOS=\left\{y|y_{i}^{min}\leq y_{i}\leq \;y_{i}^{max};1\leq i\leq p\right\}$$

Achievable Output Set (AOS): This set consists of all possible outputs that can be achieved, given the available input set and is mathematically defined as:

(11) $$AOS_{u}=\left\{y|M\left(u\right);\forall u\in AIS\right\}$$

Any steady-state operating points that lie outside of the AOS for a given design are, by definition, unachievable regardless of the control system that is ultimately selected.

Servo-OI: Without regulatory control, the servo operability index (s-OI) for this analysis is given by:

(12) $$s-OI=\frac{\mu \left(AOS\cap DOS\right)}{\mu \left(DOS\right)}$$

**Figure 4:**
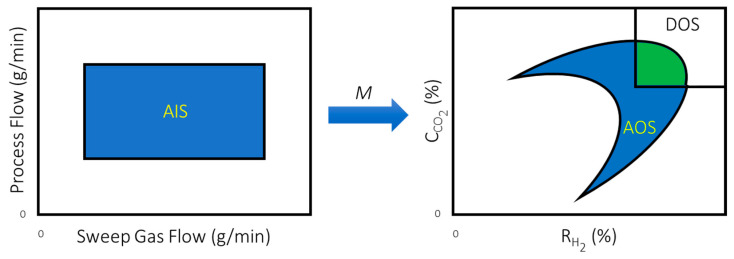
Schematic of the process operability concept. The fraction of the DOS covered by the green region would correspond to the operability index (OI) for the design.

Here, *μ* represents a measure of the size of the space, for instance length, area, volume, and hypervolume for their respective dimensions. The servo-OI is a way of quantifying what fraction of the DOS can be achieved by a given design. An OI of 1 would mean the given design can achieve any steady-state operating point in the DOS whereas an OI of 0 would mean the given design can achieve no steady-state operating points in the DOS. Figure 4 summarizes the operability concept with the green region representing the region of the DOS that can be achieved by a given design considering a schematic for the water-gas shift reaction example that will be addressed in this article [12,18].

## 3. Proposed Approach

The ultimate goal of this research is to develop an optimization algorithm that can use the novel modular design approach described above to improve the operability performance of a membrane reactor system. However, one of the biggest challenges to accomplishing this goal is the fact that there are no heuristics available for designing such systems—heuristics that, if established, could greatly reduce the complexity of a future optimization problem.

For a given membrane reactor system, there are a set of problems that here will be referred to as the “N-mod” problems. Given an objective function and a value of N, the solution to the N-mod problem is defined as the combination of N total heat exchanger, membrane separator, reactor, and membrane reactor modules that maximizes or minimizes the objective function. To study this class of problems, it is proposed using an extensive simulation approach for the 2-mod, 3-mod, and 4-mod problems since they could be solved in this way. This will allow for the studying of both how increasing the number of possible modules affects the performance of the proposed unit and gives insights into how the solutions vary as the number of modules is increased. To perform these studies for N-mod problems, the following approach detailed below is employed.

**Figure 5:**
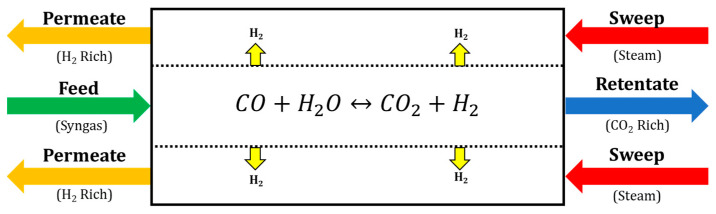
Schematic of a shell and tube WGS-MR for hydrogen recovery and carbon capture.

First, a candidate membrane reactor system for carrying out the study is selected. The selected membrane-based system is the water-gas shift, membrane reactor system (WGS-MR) developed in previous work [12,18]. This membrane reactor works by converting syngas into primarily carbon dioxide and hydrogen with the hydrogen-selective polybenzimidazole (PBI) polymer membrane removing the hydrogen as a fuel gas and leaving the carbon compounds inside the tube as shown in Figure 5. This PBI membrane was developed by Los Alamos National Laboratory to be utilized for high-temperature water-gas shift and is reported in the literature to remain stable up to temperatures of 500 °C [19,20,21] making it a promising material for future membrane reactor applications.

The reaction kinetics for this system can be described using the kinetic model proposed by Choi and Stenger [22] and is described by Equation (13).

(13) $$r_{CO}=k\left(P_{CO}P_{H_{2}O}-\frac{P_{CO_{2}}P_{H_{2}}}{K_{P}}\right)$$

in which $r_{CO}$ is the rate of consumption of carbon monoxide, k is the reaction rate coefficient, $P_{i}$ is the partial pressure of component i, and $K_{P}$ is the equilibrium coefficient for the water-gas shift reaction. The polybenzimidazole (PBI) polymer membrane’s permeation is modeled using a Fickian diffusion model as shown in Equation (14).

(14) $$J_{i}=\frac{Q_{i,o}}{\delta }\left(p_{i,t}-p_{i,s}\right)$$

where $Q_{i,o}$ is the permeance of component *i*, δ is the membrane thickness, and $p_{i,t}$ and $p_{i,s}$ are the component partial pressures on the tube and shell sides, respectively. For this work, the values of permeance come from [18] and are summarized in Table 1.

Because a polymer membrane is being utilized, other species can permeate through the membrane as well, most notably steam (which is used as sweep gas) and carbon dioxide. In the ideal case, all of the carbon-containing compounds (CO_2_ and CO) would remain on the tube side of the membrane reactor and the membrane reactor would recover all of the potential H_2_ that could be produced by the system. The performance objectives for this system can be defined by Equations (15) and (16):

(15) $$R_{H_{2}}=\frac{H_{2}\;in\;permeate}{\left(H_{2}+CO\right)in\;feed}=\frac{F_{H_{2},p}}{F_{H_{2},f}+F_{CO,f}}$$

(16) $$C_{CO_{2}}=\frac{carbon\;in\;retentate}{carbon\;in\;feed}=\frac{F_{CO,r}+F_{CO_{2},r}}{F_{CO,f}+F_{CO_{2},f}}$$

in which $R_{H_{2}}$ and $C_{CO_{2}}$ are the hydrogen recovery and carbon capture fractions, respectively. Upon closer inspection, these are competing objectives. Improved carbon capture would occur as membrane permeation in general worsens, whereas improved hydrogen recovery comes with enhanced permeation from more sweep gas being used. This means for a given flowrate of syngas on the tube side, there exists some flow rate of sweep gas that produces the “best tradeoff” between these two objectives for the nominal operating point. In this case, the best tradeoff is defined as the design and operating point that minimizes the following objective function:

(17) $$f\left(L,D,N_{t},F_{steam}\right)={\left(1-R_{H_{2}}\right)}^{2}+{\left(1-C_{CO_{2}}\right)}^{2}$$

where *L* is reactor length, *D* is the shell diameter, *N_t_* are the number of tubes, and *F_steam_* is the nominal steam sweep gas flow. This objective function is selected because a “utopian” design would give 100 percent hydrogen recovery and carbon capture, so the best tradeoff will be the design and operation that brings the membrane-based unit as close to that point as possible under normal operation. Using the sequential quadratic programming (SQP) optimization tool built into the AVEVA Process Simulation Platform, assuming 500 kg/h of syngas (modular scale) must be processed, and requiring that the tubes must be packed densely enough to assume there is no bulk diffusion, this optimal membrane design and operation was determined and its conditions are summarized in Table 2.

**Figure 6:**
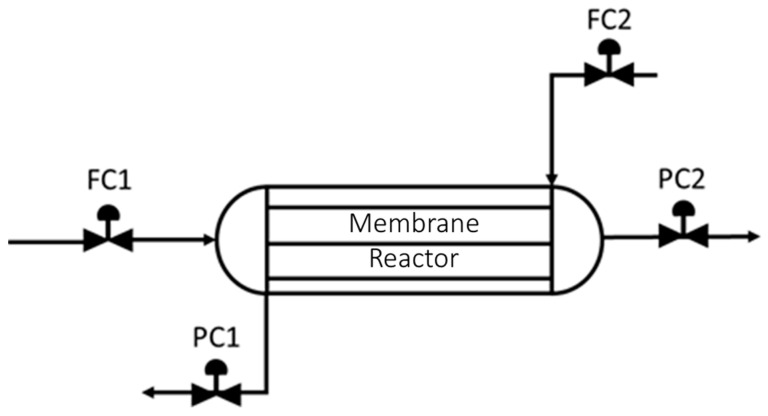
Image of the countercurrent WGS-MR setup where the syngas process flow control valve (FC1) and the sweep gas control valve (FC2) manual positions are changed to generate the AIS.

The result in Table 2 makes sense because perfect carbon capture is not achievable if there is membrane present (unless perfect selectivity is assumed); however, near perfect hydrogen recovery may be achievable by improving the operation of the membrane. Because removing membrane could improve the carbon capture percentage, this system could be a good candidate for the modular design approach described above if certain sections of the membrane reactor-based unit could have no membrane in them. The following layout in Figure 6 is initially assumed for the studies.

**Figure 7:**
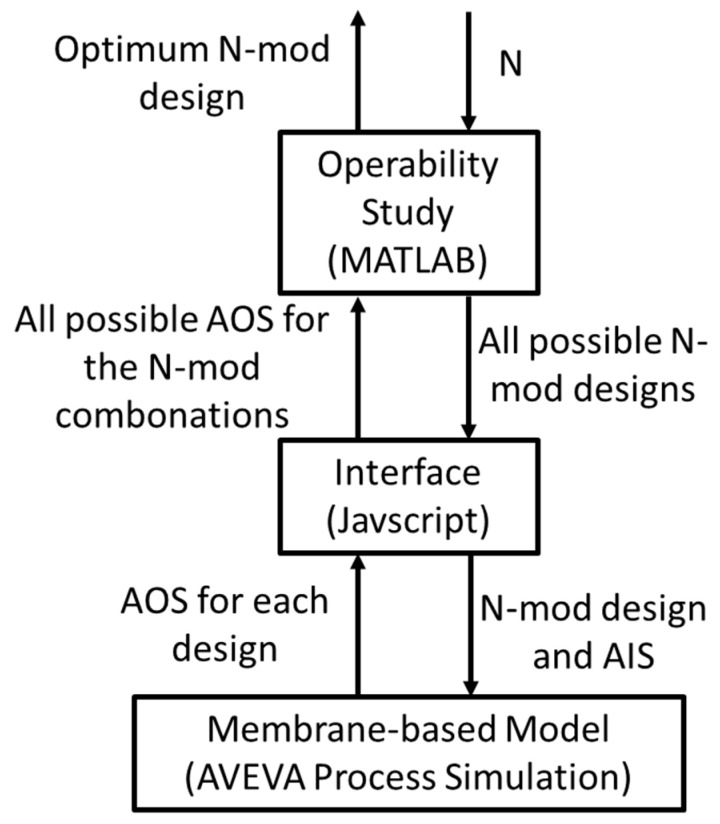
Computational framework developed for determining the optimum N-mod design.

A computational framework is then developed that allows MATLAB (where the process operability studies are performed) to send/receive values to/from the membrane model in AVEVA Process Simulation. This framework works by defining which N-mod problem is being studied, generating the design file for the membrane unit, and then sending the design to Node.js which is a platform for executing a Javascript code. Employing the AVEVA node modules developed to allow Node.js to control AVEVA Process Simulation, Node.js then takes the design from MATLAB, implements it in the simulation, performs the operability analysis, and returns the result to MATLAB. This information workflow is summarized in Figure 7.

To perform a fair study, every design is tested using identical available input sets to compare their subsequent achievable output sets. Therefore, the only change between each study is the modular design of the membrane system. An extensive simulation optimization approach is then used to guarantee that the algorithm has found the global optimum of the design space as well as allow for the opportunity to study which designs lead to more effective performance than the base case membrane reactor design.

## 4. Results

As briefly described above, the objective of this work is to investigate if using the proposed modular design approach for membrane-based systems can lead to an improvement in the operability index of the design. The OI of the membrane reactor system can be improved in two ways: (1) the area the AOS of the system coverage can be increased to overlap more of the DOS; and (2) the AOS of the system can be translated further into the DOS. To begin, it is investigated if the modular design approach can expand the size of the AOS.

It is easiest to begin with the 2-mod problem as there are only nine “viable” modular designs possible. This is because each of the two modules could be one of four possible modules: heat exchanger, reactor, membrane separator, or membrane reactor meaning there are $4^{2}$ (or sixteen) possible permutations to check. However, not all of these sixteen designs are “viable” designs. For example, one of these permutations would be two heat exchanger modules. This is not a viable design because it has no catalyst and/or no membrane present in any of the modules. Such a design defeats the purpose of process intensification and can be excluded from the extensive simulation check list. For a given N-mod problem, there are $2\times 2^{n}-1$ designs that either have no membrane or no catalyst present. This means for a given N-mod problem, the extensive simulation (or enumeration) algorithm must check the number of modules in accordance with Equation (18).

(18) $$N_{designs}=4^{N_{modules}}-2*2^{N_{modules}}+1$$

This is not a challenge for solving relatively small problems such as the 2-mod problem, but it is not a viable approach as the number of modules increases. This necessitates developing heuristics for this design approach to minimize the overall size of the problem.

**Figure 8:**
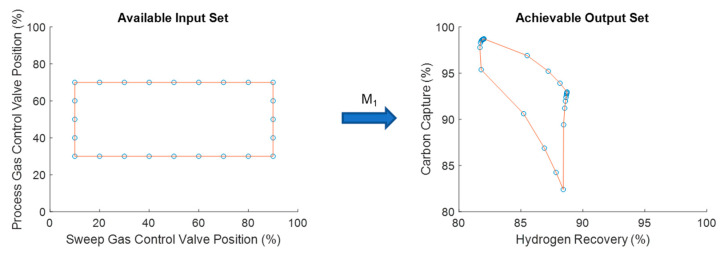
The mapping of the input space of control valve positions (AIS) to the output space of carbon capture and hydrogen recovery (AOS).

For the simulation studies, the AIS is produced by considering the combination of all possible valve positions for the process side syngas flow control valve and the shell side sweep gas flow control valve. After opening and closing these two valves, the carbon capture and hydrogen recovery rates are recorded to produce the corresponding AOS. For this case, the traditional membrane reactor design considered as the base case produces the AOS (on the right) in Figure 8 when applying the AIS (on the left) to it considering the process model, M.

**Figure 9:**
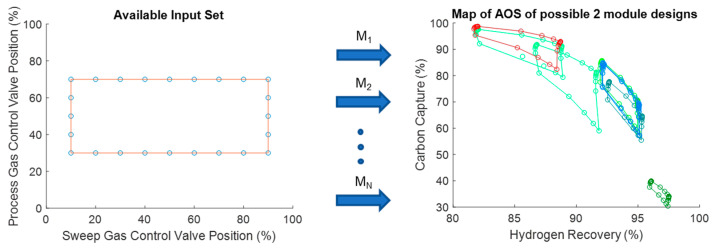
Plot showing all viable 2-mod design AOS’s given the same AIS. Here, the red AOS represents the membrane reactor AOS that is shown in Figure 8. In addition, M_1_, M_2_,… M_N_, denote the mapping of each module-based design.

By analyzing the AOS in Figure 8, the problem with membrane reactors becomes evident. It is common to see equidistant points in the AIS be mapped to a very small area of the AOS such as the points in the corners in Figure 8 (on the right). This is where the interest lies in expanding the amount of space that a given AIS is able to achieve. This process can then be repeated for every viable 2-mod designs of the membrane reactor. The results of the operability analysis for all designs are shown in Figure 9.

**Figure 10:**
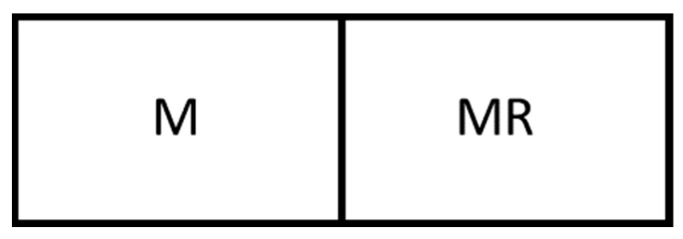
The optimum 2-mod design that maximizes the size of the AOS. In this case, this design can achieve a 65% larger space than the base case membrane reactor.

There are a few important spaces to note in Figure 9. First, some designs produce much smaller output spaces than the original membrane reactor does (AOS in red). Spaces that are much smaller than the membrane reactor original AOS represent designs that take away a hypothetical control system’s ability to affect the system. For example, the dark green AOS in Figure 9 where all points lie to the right of 95% hydrogen recovery value means that opening and closing the sweep gas and syngas control valves has little effect on the final output of that particular design. In contrast, both of the brighter green AOS sets in Figure 9 cover a larger space in area than the membrane reactor does. This means the control valves for these systems have a greater impact on the output of these designs. In this case, the modular design that maximizes the size of the AOS consists of a membrane separator and a membrane reactor and is shown in Figure 10.

**Figure 11:**
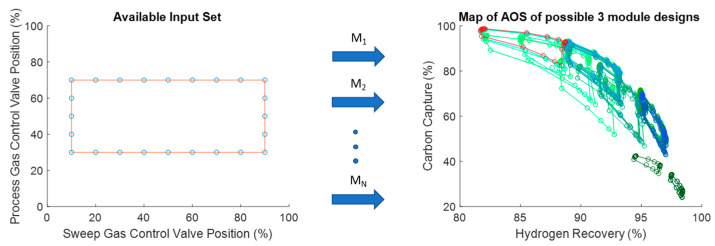
Plot showing all viable 3-mod design AOS’s given the same AIS. Here, the red AOS represents the membrane reactor AOS that was first shown in Figure 8.

This design in Figure 10 has an AOS that is 65% larger than the original AOS for the membrane-reactor design. The natural next question would be if this number could be improved by increasing the number of modules and by how much. Using the same analysis approach applied to the 2-mod problem, the output spaces to all viable 3-mod designs (49 designs in total) can be generated as shown in Figure 11.

**Figure 12:**
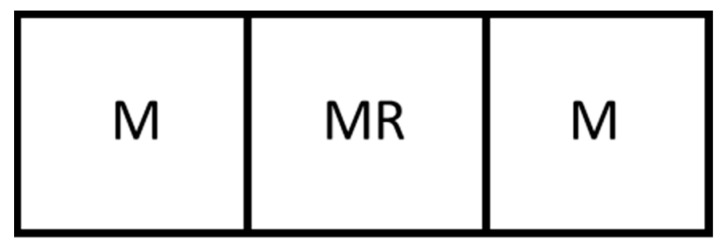
The optimum 3-mod design that maximizes the size of the AOS. In this case, this design can achieve a 67% larger space than the base case membrane reactor.

With more modules as part of the design, the number of potential AOS’s increases to 49 viable designs with similar outcomes in terms of AOS coverage to the 2-mod result. Once again, there appears to now be two designs that shrink the output space in Figure 11 when compared to a similar part of the output space rather than just one as in Figure 9. In addition, many designs seem to map to specific areas of the output space. This suggests that there are certain sets of designs that belong to the same “family” (represented by the light green AOS sets in Figure 11) in the sense that they map the input set to the output set in similar ways. For the 3-mod problem, the optimum design was able to increase the size of the AOS by 67% compared to the original membrane reactor base case which is shown in Figure 12.

**Figure 13:**
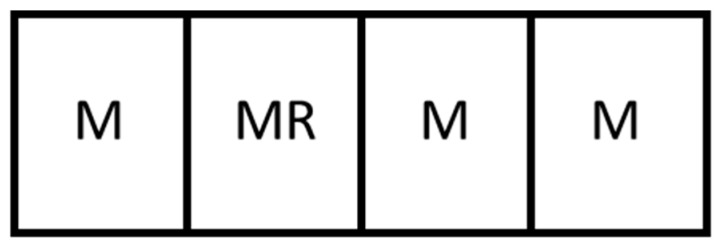
The optimum 4-mod design that maximizes the size of the AOS. In this case, this design can achieve a 76% larger space than the base case membrane reactor.

This result corresponds only to a small increase when compared to the 2-mod’s solution that increased the AOS’s area by 65%. An interesting observation to make is to compare the 2-mod and 3-mod solutions. The solutions are similar in their structures and the 3-mod solution only differs by including a membrane module attached to the right side of the arrangement. Continuing this process for the 4-mod problem, an optimum solution is obtained which increases the AOS area by 76%, a much larger jump than before, which is shown in Figure 13.

The observation of the optimum solution for each N-mod problem being similar to the previous solution appears to be becoming a trend. This trend is expected as the global optimum should not drastically switch for optimal performance. This solution also makes sense for why it is so effective at expanding the output space. The initial syngas that enters the unit contains some amount of hydrogen. By utilizing a membrane separator first, this allows the control valves to control the amount of this initial hydrogen that is removed before reaction. This ultimately gives control of the degree for which the water-gas shift reaction occurs as more or less hydrogen being present in the membrane reactor section would ultimately control the reaction rate and equilibrium. Then the remaining two membrane modules can remove whatever amount of hydrogen is left after the reaction. Thus, this resulting design is still experiencing the benefits from process intensification, but it now behaves more like a traditional unit operation approach would from a control standpoint. It should be noted that when comparing these optimal solutions, the total length of the module-based design is held constant whereas the individual modules are shortened to fit within that specified total length. A summary of the 2-mod, 3-mod, and 4-mod solutions is provided in Table 3.

Unfortunately, this process must stop here because the size of the problem grows too large when moving to the 5-mod problem (approximately 3.76 times more possible designs than the 4-mod problem). However, an argument could now be made that not all designs need to be checked to find an optimum solution for higher numbers of modules. For the sake of argument, one could imagine that there exists a 100-mod solution. Although these modules would be incredibly thin and probably not economically feasible to construct, a theoretical solution would still exist. If then the 101-mod solution was desired, would it be expected the answer would be almost identical to the previous one? Each module would almost be the exact same length in almost the same locations under the same conditions. The only problem is that there is one additional module that needs to be assigned. For higher values of N, then, it is a safe assumption that the optimum solutions by one module. That is to say, the previous (N-1)-mod solution is generally a subset of the optimum permutation of the following N-mod problem. For example, the 3-mod solution in Table 3 consists of the 2-mod solution plus an additional membrane separator and the 4-mod solution consists of the 3-mod solution plus a membrane separator.

Next, one can also explore if it is possible to translate the AOS of a module-based design closer to the DOS than the original membrane reactor. To perform this task, the same process is followed as before, however, the best design is defined as the design that minimizes the Euclidean distance between the points in the AOS and the utopian point of 100% hydrogen recovery and carbon capture. A summary of the results for this analysis is shown in Table 4.

These results seem to not be as impressive as the ones for expanding the AOS’s size, but this is expected. Recall that the original membrane reactor design was chosen through an optimization that was minimizing the Euclidean norm between the nominal operating point and the utopian point in Equation (15). So, although the changes are small, it is still a positive outcome that this modular approach produced designs that improved this objective over the original membrane reactor. In addition, notice that the pattern of solution similarities is also observed for this objective function as well.

**Figure 14:**
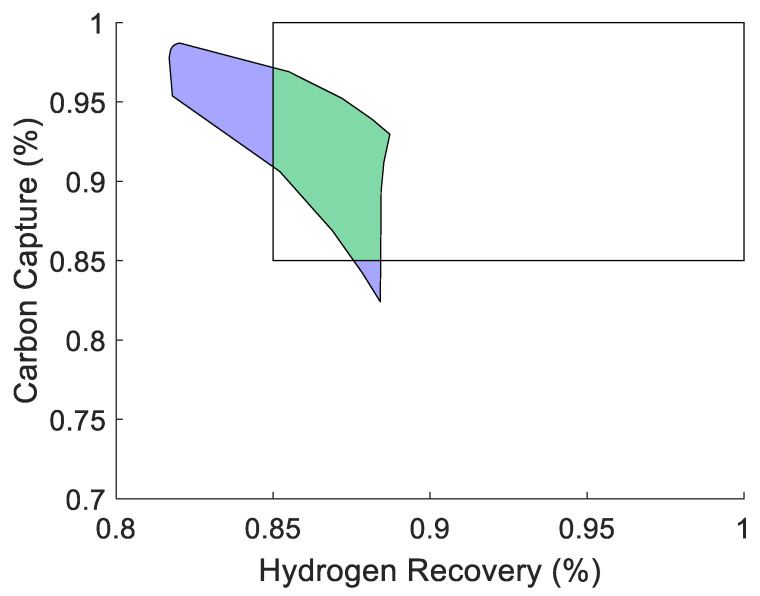
The AOS of the original membrane reactor is shown (in blue/green). The area of the overlap (green) with the DOS (white) has an OI of 0.129 meaning the membrane reactor can achieve 12.9% of the DOS.

Thus, these analyses demonstrate that the proposed novel modular design approach for membrane reactor systems can both expand the size of the AOS and translate it closer to the utopian point, and therefore, further into the DOS. This suggests that this design approach should be effective in improving the operability index for membrane-based systems. For this analysis, a DOS is chosen that includes all operating points with a hydrogen recovery and carbon capture greater than or equal to 85% as a value of 90% is not achievable for both parameters simultaneously based on the previous studies. To determine the operability index for each design, the “boundary”, “polyshape”, and “intersect” functions in MATLAB were used. These functions allow for the identification of the boundary points of the AOS, convert it to a polygon object, and then measure the area enclosed by the boundaries and calculate the area of overlap, respectively. An example of a result for this analysis is show in Figure 14.

This process was then repeated for each modular design of the 2-mod, 3-mod, and 4-mod problems and the results are summarized in Table 5.

**Figure 15:**
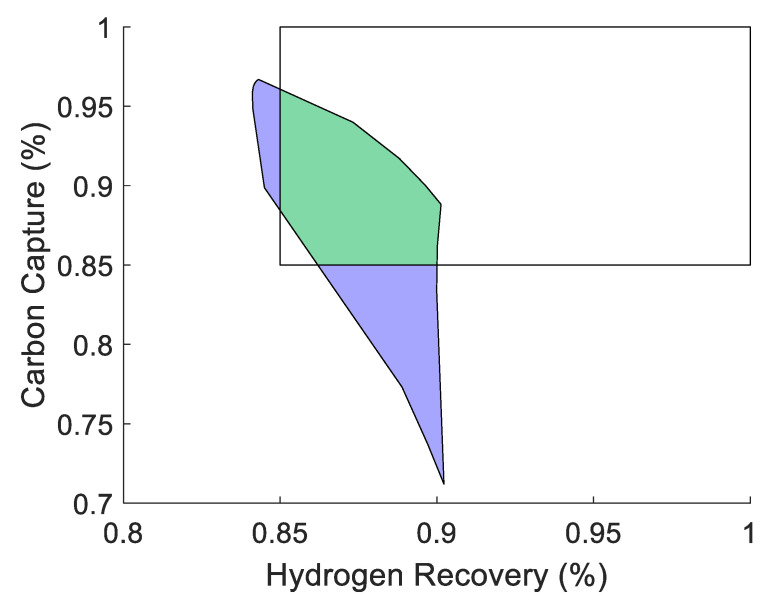
The AOS of the optimum 4-mod design is shown (in blue/green). The area of the overlap (green) with the DOS (white) has an OI of 0.176, approximately a 37% improvement from the base case.

To show this improvement, the result of the operability analysis for the 4-mod problem which led to the greatest improvement in the servo-OI from the base-case membrane reactor, is shown in Figure 15. This shows how the AOS for the optimum 4-mod design is able to cover more of the DOS and therefore, has a larger servo-OI.

This result shows that the proposed modular design approach is capable of improving the operability of the membrane-based system. With the 4-mod design shown in Table 5, approximately 37% more of the DOS is now achievable regardless of the control structure applied to the system when compared to what the original membrane reactor is capable of. However, the trend of similar solutions from one N-mod problem to the next seen in the previous optimizations no longer appears to be true. As stated previously, maximizing the OI can be done by expanding the size of the AOS and/or translating the space further into the DOS. Table 3 showed that as the number of modules increases, the improvement in the size of the AOS area from one global optimum to the next grows monotonically. However, this was not the case for the translation analysis from Table 4. It appears likely that when the objective function is switched to the OI, a combination of these two observations explains why increasing the number of modules can lead to large improvements, but not monotonically and not with similar solutions’ nature.

This observation has major implications for the development of future optimization formulations. If the main focus was to either improve the AOS area or minimize the distance between the AOS and the DOS, then using the solution similarity assumption greatly reduces the problem size. As each solution would only differ by one module, the number of designs that need to be checked in an extensive simulation approach dramatically decreases. Rather than the problem growing exponentially, it now grows by the following rate:

(19) $$N_{designs}\leq \frac{3}{2}n^{2}{}_{modules}+\frac{5}{2}n^{2}{}_{modules}-4$$

In this case, it is less than or equal to the rate in Equation (19) because there is a chance that designs that are in the neighborhood of the initial guess could be invalid designs and therefore do not need to be checked. This means for the optimizations shown in Table 3 and Table 4, the 12-mod problem could be solved in the same amount of time as the 4-mod problem if all viable designs are checked. However, since this does not apply to the servo-OI, other techniques must still be developed to reduce the time required to optimize for the servo-OI. The hope would be that since improving the OI involves both this expansion and translation process, that the heuristics identified for these processes can be combined to develop a heuristic approach to improving the OI of membrane reactor systems.

## 5. Conclusions

One of the ways to intensify a conventional unit operation is to combine phenomena into a single unit. To understand the problem further, a novel module-based membrane reactor design approach is proposed where smaller, identically sized modules are constructed. To investigate this design approach’s impact on the DOF reduction challenge, an operability framework was applied to this modular membrane reactor design approach for the first time. Other work in the literature has employed the concept of flexibility in the design of reactive separation systems, but the operability index was used in this work as a more comprehensive measure of controllability and flexibility of a design and control system. This is because an improvement in the operability index implies a control system is capable of achieving more desired operating points given then same set of available control moves.

The performed operability analysis led to a few important contributions in this area of research. First, the approach to designing membrane reactors can significantly increase the size of the AOS and thus can provide a given set of control valves far more freedom to achieve a desired output. Second, this approach to design can also translate the AOS closer to the DOS, even when the original membrane reactor design was optimized for this purpose. Third, when trying to improve one of these objectives, the global optimum for each N-mod problem (defined as the optimum arrangement of N number of phenomena-based modules in a membrane reactor system) appears to be in the neighborhood of the previous (N-1)-mod solution. This observation can be used to greatly reduce the size of the problem and allow an extensive simulation approach to be tractable. Lastly, this design approach can improve the operability index as compared to the original membrane reactor design; however, the solution similarity observation does not appear to apply for this objective. The observations in this work will greatly assist going forward in developing a general optimization approach for maximizing the operability index using this module-based membrane approach.

Designing membrane reactor systems in this way comes with many potential benefits. A membrane reactor on its own experiences a loss in degrees of freedom which is only made worse by the fact that it must service multiple sites with varying feed conditions. Currently, however, this is the most economical way to approach the intensification of membrane reactor systems because mass producing one membrane reactor design would yield more economic benefits than manufacturing a unique design for multiple sites. By adopting the module-based design approach discussed in this work, individual modules could be mass produced to capitalize on the economic benefit, and then customized and fitted for each site’s specific operational needs. As shown in the Results section of this work, improved overall performance would be expected as well by allowing for a module-based design of membrane reactors.

Although this work has shown that a hybrid design approach, rather than a pure membrane reactor or a purely unit operations-based design approach (with membrane and reactors used in separate), can lead to better operability and performance; this work does not identify a computationally efficient method for finding the solution. Currently there are two limitations with this method: (1) it requires a separate piece of software to facilitate the communication between the algorithm in MATLAB and the simulation in AVEVA Process Simulation; (2) although there is a simple way for drastically reducing the number of designs to check when moving from the N-mod to the (N+1)-mod problem for maximizing the operability index or the performance, this observation does not seem to hold when performing a multi-objective optimization involving the two. This means (a) that our findings show that an exhaustive simulation approach is the only currently available way of maximizing both performance indices and (b) that either (1) a similar heuristic needs to be discovered for the multi-objective case or (2) an alternative optimization approach is required for cases with more modules. Both of these directions are being investigated as future work.

## Figures and Tables

**Table 1:** Component permeance (*Q_i,o_*) values used reported in gas permeation units (GPU).

Component (i)	*Q_i,o_* (GPU)
H_2_	250.0
CO_2_	8.9
H_2_O	750.0
CO	2.5
N_2_	2.5

**Table 2:** Optimal membrane reactor design that produces the “best tradeoff” between hydrogen recovery and carbon capture according to the defined objective in Equation (17).

**Parameter**	**Optimal Value**
L	4.9 m
D (shell)	3.3 m
Nt	53
Fsteam	1.88 kg/h
$R_{H_{2}}$	0.949
$C_{CO_{2}}$	0.870

**Table 3:** Summary of the operability analyses for maximizing the AOS area.

N	Possible Designs	Optimum Design (for Maximizing the Servo-OI)	Increase in the OI Compared to the Membrane Reactor Base Case
2	9	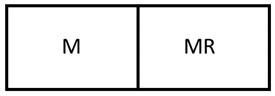	+65%
3	49	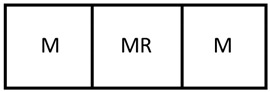	+67%
4	255	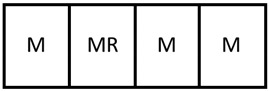	+76%

**Table 4:** Summary of the operability analyses for minimizing the Euclidean distance between the points in the AOS and the utopian point of 100% hydrogen recovery and carbon capture.

N	Optimum Design (for Decreasing Euclidian Distance yo Utopian Point)	Decrease in the Euclidean Distance of the Aos and the Utopian Point
2	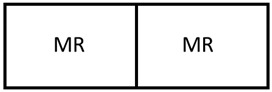	0%
3	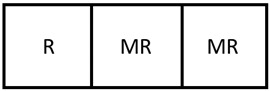	−3%
4	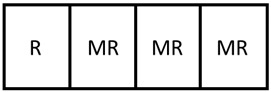	−0.3%

**Table 5:** Summary of the operability and optimization analyses for maximizing the servo-OI.

N	Optimum Design (for Maximizing the Servo-Oi)	Increase in the Oi Compared to the Membrane Reactor Base Case
2	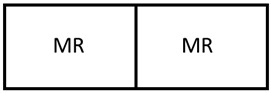	+12%
3	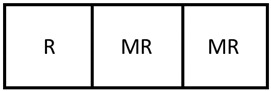	+9%
4	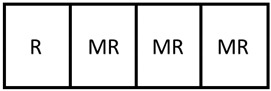	+37%
